# FGF/MAPK signaling sets the switching threshold of a bistable circuit controlling cell fate decisions in embryonic stem cells

**DOI:** 10.1242/dev.127530

**Published:** 2015-12-15

**Authors:** Christian Schröter, Pau Rué, Jonathan Peter Mackenzie, Alfonso Martinez Arias

**Affiliations:** Department of Genetics, University of Cambridge, Downing Street, Cambridge CB2 3EH, UK

**Keywords:** Embryonic stem cells, GATA factor, Cell fate decision, Dynamic systems, Live cell imaging, Preimplantation development

## Abstract

Intracellular transcriptional regulators and extracellular signaling pathways together regulate the allocation of cell fates during development, but how their molecular activities are integrated to establish the correct proportions of cells with particular fates is not known. Here we study this question in the context of the decision between the epiblast (Epi) and the primitive endoderm (PrE) fate that occurs in the mammalian preimplantation embryo. Using an embryonic stem cell (ESC) model, we discover two successive functions of FGF/MAPK signaling in this decision. First, the pathway needs to be inhibited to make the PrE-like gene expression program accessible for activation by GATA transcription factors in ESCs. In a second step, MAPK signaling levels determine the threshold concentration of GATA factors required for PrE-like differentiation, and thereby control the proportion of cells differentiating along this lineage. Our findings can be explained by a simple mutual repression circuit modulated by FGF/MAPK signaling. This might be a general network architecture to integrate the activity of signal transduction pathways and transcriptional regulators, and serve to balance proportions of cell fates in several contexts.

## Abstract (precis)

**Highlighted article:** Quantitative measurements and mathematical modeling reveal the integration of transcription factor activity and extracellular signaling during fate decisions in ESCs.

## INTRODUCTION

To ensure the faithful development of multicellular organisms, cell fate decisions in populations of undifferentiated cells have to be tightly balanced. It is now well established that transcriptional networks and extracellular signals together control these decisions, but how their interactions determine the proportions of cells differentiating along particular lineages is often not known. This question is of particular importance in one of the first cell fate decisions in the mammalian preimplantation embryo, where a small number of inner cell mass (ICM) cells have to reliably populate both the epiblast (Epi) lineage that will give rise to the embryo proper, as well as the primitive endoderm (PrE) lineage, which differentiates into tissues that function in patterning and nutrient supply of the embryo (Rossant and Tam, 2009). The factors underlying this fate decision have been studied in embryos and embryonic stem cells (ESCs) (Cho et al., 2012; Fujikura et al., 2002; Shimosato et al., 2007), clonal derivatives of ICM cells that are biased towards the Epi fate but harbor a latent PrE differentiation potential (Beddington and Robertson, 1989; Morgani et al., 2013). Mutant analysis indicates that a transcriptional network centered on the transcription factor NANOG marks and defines the Epi fate (Chambers et al., 2007; Frankenberg et al., 2011), whereas a network centered on GATA transcription factors underlies the PrE fate (Bessonnard et al., 2014; Schrode et al., 2014). NANOG and GATA6 are co-expressed in early ICM cells, but their expression patterns become mutually exclusive as cells commit to specific fates (Plusa et al., 2008), suggesting mutually repressive interactions between the two programs (Frankenberg et al., 2011; Schrode et al., 2014). Studies using genetic mutants and pharmacological inhibitors have furthermore shown that the FGF/MAPK signaling pathway promotes the PrE fate at the expense of the Epi fate in embryos (Kang et al., 2012; Nichols et al., 2009; Yamanaka et al., 2010), and that it is required for PrE-like differentiation of ESCs (Cho et al., 2012). How the activities of the transcriptional networks are integrated with the activity of the FGF/MAPK signaling pathway, and how these inputs together control the proportion of cells differentiating along either lineage has not been systematically investigated.

Recently, a mathematical model for the decision between the Epi and the PrE fate has been proposed (Bessonnard et al., 2014), in which *Nanog* and *Gata6* repress each other, and reinforce their own expression through direct positive feedback. This defines a dynamic system with three stable states in which cells either express GATA6 or NANOG alone, or co-express the two markers. In this model, FGF/MAPK signaling both promotes GATA6 expression and inhibits NANOG expression, and differences in FGF/MAPK signaling between cells have been proposed to underlie fate choice from the co-expression state (Bessonnard et al., 2014). Although this model is consistent with static phenotypes of wild-type embryos and genetic mutants, the gene expression dynamics proposed have not directly been tested. It is also not clear whether all proposed links are required to explain the behavior of the genetic circuit underlying this cell fate decision, and which one of the two inputs into the system – signaling or transcription factor activity – most influences the fate decision. Addressing these open questions requires quantitative modulation of the inputs into the genetic circuit regulating fate choice, and following its dynamics in single cells in real time. Here, we achieve this by transiently expressing fluorescently tagged GATA factors in ESCs carrying live reporters for the Epi and the PrE fate. This allows us to recreate a state of co-expression of Epi and PrE determinants akin to the state of ICM cells in the embryo, and to follow the resolution of this state in real time. We find that cells rapidly exit the co-expression state towards one of two mutually exclusive states, i.e. the system is bistable. PrE-like differentiation occurs in cells exposed to GATA factor levels above a threshold, and the function of FGF/MAPK signaling is to set this threshold dose. This provides a mechanism through which both transcription factor activity and signaling can tune the proportions of cells with specific fates. Recapitulating the dynamic behavior of the circuit *in silico* only requires mutual repression between the transcriptional networks underlying the Epi and the PrE fates without any positive feedback loops, and a single repressive input of MAPK signaling on the Epi-specific program. This data-based model for the Epi-versus-PrE fate decision, much simpler than previously proposed models, will serve as a basis to guide further experimental and theoretical exploration of this crucial fate decision of mammalian embryogenesis. Furthermore, our finding that FGF/MAPK signaling can balance the proportions of alternative fates in cell populations by setting the response threshold of a regulatory network to a transcription factor input is a novel principle for this signaling pathway which might be relevant in developing tissues beyond the ICM.

## RESULTS

### An ESC model system to investigate PrE-like fate choice in culture

**Fig. 1.**
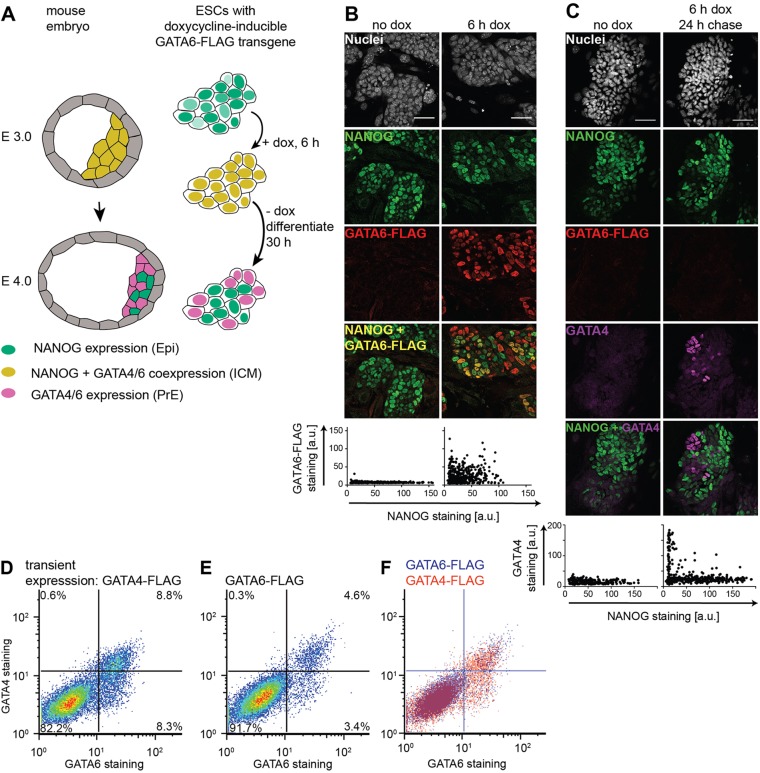
**Expression of endogenous markers of PrE-like differentiation following transient expression of GATA6-FLAG and GATA4-FLAG.** (A) Experimental approach. Doxycycline-induced transgene expression creates a GATA6/NANOG co-expression state in ESCs similar to the situation in the ICM, from which cells can embark on PrE-like differentiation, or return to the NANOG-positive state. (B) Immunostaining (upper panel) and quantification (lower panel) of untreated (left) or doxycycline-treated (right) inducible ESCs indicates co-expression of NANOG and GATA6-FLAG in individual cells after 6 h of doxycycline treatment. Co-expression is limited because of heterogeneous NANOG and GATA6-FLAG expression in the presence of serum and feeders. (C) Immunostaining (upper panels) and quantification (lower panels) of NANOG and GATA4 expression 24 h after the end of a 6 h doxycycline pulse. GATA4 expression depends on doxycycline treatment, and is mutually exclusive with NANOG expression. (D-F) Flow cytometry of cells immunostained for endogenous GATA4 and GATA6 one day after transient GATA4-FLAG (D) or GATA6-FLAG (E) expression. (F) Overlay of D and E. Scale bars: 50 µm.

To model in culture the transition from GATA6/NANOG co-expression to mutually exclusive expression of Epi and PrE markers that characterizes the Epi-versus-PrE fate decision (Plusa et al., 2008), we used a doxycycline-inducible system to transiently express GATA6-FLAG in ESCs (Beard et al., 2006; Mulvey et al., 2015; Wamaitha et al., 2015) (Fig. 1A). Individual cells co-expressed inducible GATA6-FLAG and endogenous NANOG protein after a 6 h doxycycline pulse (Fig. 1B). Twenty-four hours after doxycycline removal, the cells had degraded the exogenous GATA6-FLAG, but a subset now stained positive for the endogenous PrE marker GATA4 (Fig. 1C). Virtually all GATA4-positive cells were negative for NANOG staining, suggesting that following GATA6/NANOG co-expression, ESCs transition to one of two mutually exclusive states, marked by the expression of Epi and PrE markers, respectively. This is similar to the behavior of ICM cells, and suggests that a previously reported stable state of co-expression of NANOG and endogenous GATA factors (Bessonnard et al., 2014) is not accessible in our system.

Consistent with previous studies (Fujikura et al., 2002; Mulvey et al., 2015; Shimosato et al., 2007), we found that transient expression of doxycycline-inducible GATA4-FLAG instead of GATA6-FLAG led to the same expression pattern of endogenous GATA factors, but doubled the proportion of differentiating cells (Fig. 1D-F, Fig. S1). This led us to induce PrE-like differentiation with GATA4 and to use endogenous GATA6 expression to monitor the differentiation event in all following experiments. Furthermore, we tagged the inducible GATA4 protein with an mCherry fluorescent protein. This did not compromise the activity of the fusion protein to induce PrE-like differentiation (Fig. S2), and allowed us to follow the heterogeneous expression of the doxycycline-induced transgene in individual live cells.

### ESC culture conditions affect the expression of endogenous PrE markers following a GATA4-mCherry pulse

**Fig. 2.**
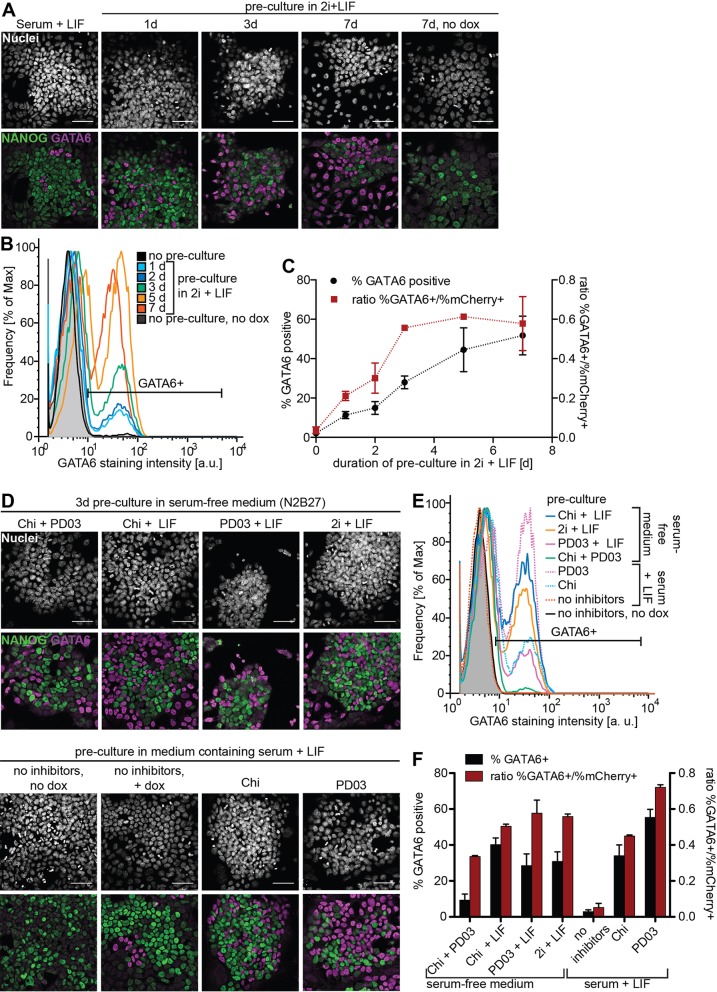
**Culture conditions affect responsiveness to GATA4-mCherry expression.** (A) Immunostaining of ESCs cultured for indicated times in 2i+LIF before a 6 h pulse of GATA4-mCherry expression followed by a 24 h chase in medium containing serum+LIF. (B) Flow cytometry of cells treated as in A and stained for GATA6. (C) Percentage of GATA6-positive cells (black) and ratio of the percentages of GATA6-positive and mCherry-positive cells (red) for different durations of pre-culture in 2i+LIF. Data averaged from three (% GATA6-positive) or two (ratios) independent experiments, errors bars state s.d. (D) Immunostaining of ESCs cultured for 3 days in the indicated media before a 6 h pulse of GATA4-mCherry expression followed by a 24 h chase in medium containing serum+LIF. Chi, CHIR99021. (E) Flow cytometry of cells treated as in D stained for GATA6. (F) Average percentage of GATA6-positive cells (black) and ratio of the percentages of GATA6-positive and mCherry-positive cells (red) for different pre-culture media. Data averaged from three (% GATA6-positive) or two (ratios) independent experiments, errors bars indicate s.d. Scale bars: 50 µm.

For an induced transcription factor to trigger a specific differentiation program, this program needs to be molecularly accessible. We therefore set out to determine culture conditions for which transient GATA4-mCherry expression led to efficient expression of endogenous GATA6. In the presence of feeders and 15% serum, a 6 h pulse of GATA4-mCherry expression resulted in ∼10% GATA6-positive cells 24 h later. This proportion dropped to ∼1.5% GATA6-positive cells when cells were cultured without feeders in 10% serum (Fig. S3A,B), even though GATA4-mCherry was efficiently induced in both conditions (Fig. S3C) and cells were positive for the pluripotency marker NANOG (Fig. S3A). Next, we pre-cultured cells in 2i+leukemia inhibitory factor (LIF), a condition reported to promote extraembryonic differentiation potential (Morgani et al., 2013), before simultaneous addition of doxycycline and transfer into serum-containing medium. This increased the proportion of GATA6-positive cells induced by a 6 h doxycycline pulse from 11.3±1.8% (mean±s.d.) for 1 day of pre-culture in 2i+LIF to 51.7±9.8% for 7 days of pre-culture (Fig. 2A-C). Because the duration of the pre-culture in 2i+LIF also affected the levels of GATA4-mCherry expression induced by doxycycline (Fig. S4A), we determined the ratio between the fraction of GATA6-positive cells one day after a 6 h doxycycline pulse and the fraction of GATA4-mCherry-positive cells immediately after the pulse as a measure for the efficiency of PrE-like differentiation. This ratio plateaus at ∼55% after 3 days of pre-culture in 2i+LIF (Fig. 2C).

To assess the influence of the components of 2i+LIF, we removed each of them from the complete 2i+LIF medium or added them individually to serum-containing medium during 3 days of pre-culture. All conditions led to an increase in the percentage of GATA6-positive cells 24 h after a 6 h doxycycline pulse, albeit to different degrees (Fig. 2D-F). The largest proportion of GATA6-positive cells was obtained for pre-culture in serum, LIF and the MEK inhibitor PD0325901 (PD03) (Fig. 2F, Fig. S4B). We conclude that inhibition of MAPK signaling prior to the induced expression of GATA factors efficiently restores PrE-like differentiation potential in ESCs. For all following experiments, we therefore pre-cultured cells for 3 days in the presence of PD03 in medium containing serum and LIF.

### Transient expression of exogenous GATA4-mCherry induces stable PrE-like differentiation

**Fig. 3.**
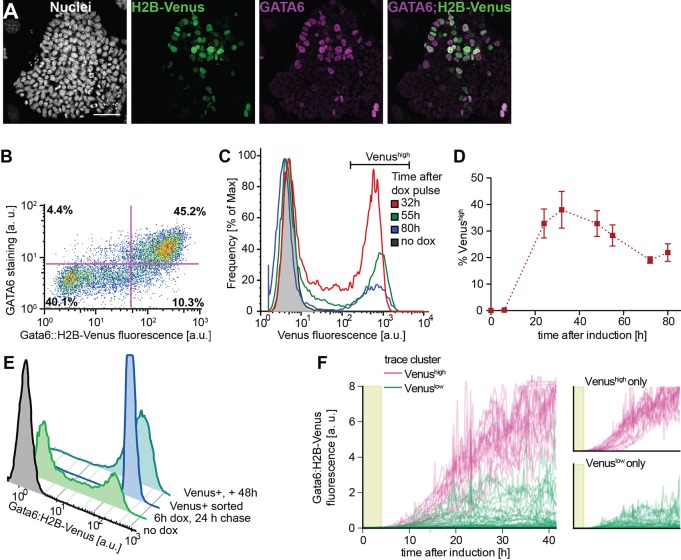
**Transient GATA4-mCherry expression induces stable expression of PrE marker genes.** (A) Immunostaining for Venus and GATA6 protein in Gata6:H2B-Venus reporter cells 24 h after a 6 h pulse of doxycycline-induced GATA4-mCherry expression. Scale bar: 50 µm. (B) Flow cytometry of GATA6-reporter cells treated as in A. (C) Flow cytometry detecting Gata6:H2B-Venus expression at indicated time-points after a 6 h pulse of GATA4-mCherry induction. (D) Percentages of Venus^high^ cells at different times after a 6 h doxycycline pulse. Data points represent mean±s.d. from three independent experiments. (E) Flow cytometry of Venus^high^ cells sorted 18 h after the end of a 6 h doxycycline pulse (royal blue), cultured for 48 h and analyzed for Venus expression. (F) Venus fluorescence intensity values of individual GATA6-reporter cells tracked in time-lapse movies during and following a 6 h doxycycline pulse (light green shaded area). Color-code is informed by hierarchical clustering based on Venus expression levels. Small panels on the right show traces for each cluster separately. See also Fig. S6 and Movie 1.

Having established an experimental regime which induced PrE-like differentiation in ESCs with an efficiency mimicking PrE differentiation in the embryo (Schrode et al., 2014), we next wanted to investigate the stability of the GATA6-positive state and the dynamics with which it evolved. To this end we created a Gata6:H2B-Venus transcriptional reporter (Freyer et al., 2015) in cells carrying the inducible GATA4-mCherry transgene, which faithfully recapitulated GATA6 protein expression between one and three days after the doxycycline pulse (Fig. 3A,B; Fig. S5). Transient GATA4-mCherry expression led to a characteristic bimodal distribution of Gata6:H2B-Venus expression (Fig. 3C). Venus expression levels of cells in the Venus^high^ peak were constant between 32 h and 80 h after the end of a 6 h doxycycline pulse (Fig. 3C,D). Furthermore, whereas cells with intermediate Venus levels progressively disappeared from the distribution (Fig. 3C), cells sorted for highest Venus expression levels maintained their fluorescence intensity over several cell divisions for 48 h (Fig. 3E). Together, these findings indicate that strong reporter expression marks a stable state in individual cells, and suggests that the decrease in the proportion of Venus^high^ cells is mainly a result of reduced proliferation of the Venus^high^ cells compared with the Venus^low^ cells, although we cannot rule out that undifferentiated cells induce reversion of Venus-positive cells in unsorted populations. Finally, to follow the dynamics of Gata6:H2B-Venus expression over time in individual cells, we performed time-lapse microscopy and tracking of reporter cells (Movie 1). Clustering of traces according to H2B-Venus expression levels identified two distinct classes of cells (Fig. 3F, Fig. S6). Expression traces corresponding to these two classes were separated throughout the experiment. Whereas some cells with intermediate Venus levels reverted to a Venus-negative state, consistent with the depletion of this population that we had observed by flow cytometry, cells with highest Venus expression remained in this class throughout the experiment (Fig. 3F). These results show that a transient GATA4-mCherry input elicits stable expression of one of two mutually exclusive expression programs, suggesting the system behaves as an irreversible switch with two stable states.

### A threshold level of GATA4-mCherry controls PrE-like differentiation

**Fig. 4.**
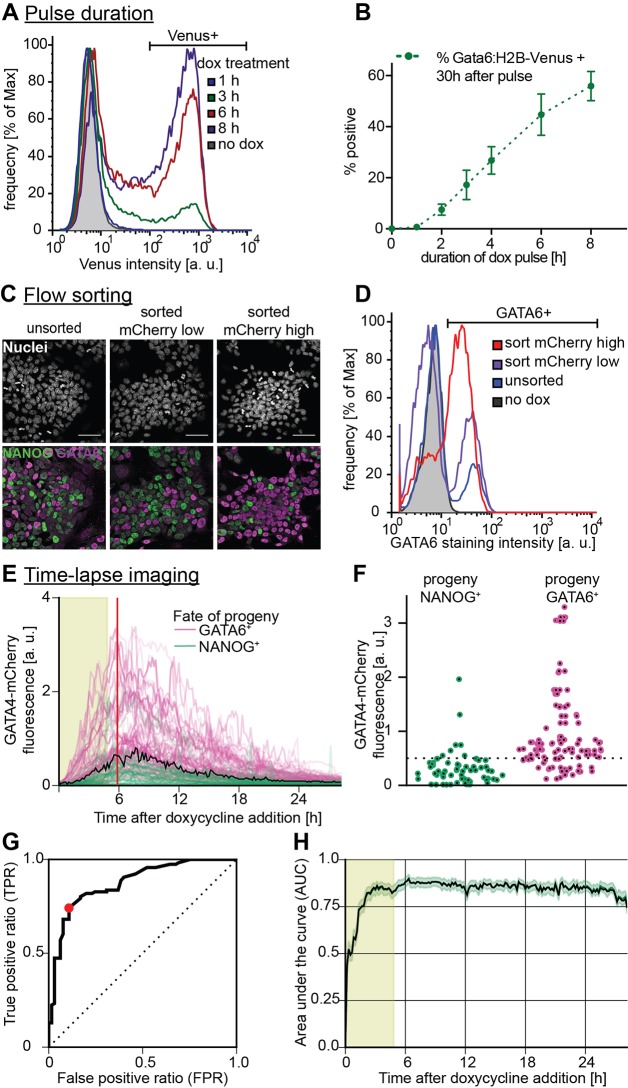
**A GATA4-mCherry threshold dose determines PrE-like differentiation.** (A) Flow cytometry for Gata6:H2B-Venus fluorescence one day after transient GATA4-mCherry expression triggered by doxycycline pulses of indicated lengths. (B) Quantitative analysis of results from A. Data points show mean±s.d. from three independent experiments. (C) Cells sorted for low (middle) and high (right) GATA4-mCherry expression levels after a 6 h doxycycline pulse and immunostained 30 h after re-plating. Unsorted control is on the left. Scale bar: 50 µm. (D) Flow cytometry of cells treated as in C stained for GATA6 expression. Purification increases the proportion of differentiating cells in both sorted pools compared with the unsorted control, and a larger proportion of GATA4-mCherry^high^ cells activate GATA6 expression compared with GATA4-mCherry^low^ cells. (E) GATA4-mCherry fluorescence traces of cells filmed during and after a 6 h doxycycline pulse. Color-code of individual traces is informed by immunostaining for NANOG and GATA6 at the end of the time-lapse. Area shaded in light green indicates duration of doxycycline pulse, red bar indicates time point analyzed in F,G, and black curve indicates optimal threshold calculated by ROC. See also Movie 2 and Fig. S9. (F) GATA4-mCherry fluorescence intensity of cells from the experiment shown in E at a single time-point (red vertical line in E). Optimal threshold to predict fate choice estimated by ROC analysis is indicated by a black dotted line. (G) ROC curve for the time-point shown in F. The optimal threshold maximizing the difference between the true positive and false positive prediction rate (TPR and FPR) is indicated by a red dot. (H) Area under the curve (AUC) values from ROC analysis in all time frames of the experiment shown in E. Error margin indicates s.d. determined by bootstrapping (*n*=1000).

We then asked whether the flipping of the bistable switch that we had identified depended on the expression levels of the doxycycline-induced GATA4-mCherry protein. Varying the duration and levels of GATA4-mCherry exposure by applying doxycycline pulses of different lengths (Fig. S7) smoothly tuned the proportion of Gata6:H2B-Venus^high^ cells (Fig. 4A,B). Furthermore, we observed more differentiating GATA6-positive cells and fewer undifferentiated NANOG-positive cells in populations that had been sorted for high GATA4-mCherry expression levels immediately after the doxycycline pulse compared with populations sorted for low GATA4-mCherry expression (Fig. 4C,D; Fig. S8). Together, this suggests that GATA4-mCherry expression levels control the proportion of cells undergoing PrE-like differentiation.

To correlate GATA4-mCherry input levels more precisely with subsequent fate choice in single cells, we performed time-lapse imaging of GATA4-mCherry-inducible cells during and after a doxycycline pulse, followed by immunostaining for NANOG and GATA6 (Movie 2; Fig. S9A,B). We found that most cells with GATA6-positive progeny had experienced higher GATA4-mCherry expression levels than cells with NANOG-positive progeny (purple and green datapoints in Fig. 4E,F and in Fig. S9C). We used receiver operating characteristics (ROC) analysis (Fawcett, 2006) (for details see the supplementary materials and methods) to assess how well the two classes of cells could be separated by a threshold value of GATA4-mCherry expression. Plotting the ratio of correctly and incorrectly separated events over the total number of cells (true positive ratio, TPR; false positive ratio, FPR) for varying threshold values gives a characteristic curve for a single time-point (Fig. 4G); the larger the area under this curve (AUC), the better the differentiation outcomes can be separated or predicted from GATA4-mCherry expression levels. The AUC increased quickly upon doxycycline addition and reached a plateau between 0.8 and 0.9 after ∼3 h (Fig. 4H). Similar results were obtained when we used cumulative instead of instantaneous GATA4-mCherry expression measurements (not shown), suggesting that non-systematic measurement errors are not a major limitation of predictive power. The optimal prediction threshold that maximizes the difference between TPR and FPR tracked the expression dynamics of the GATA4-mCherry protein throughout the experiment (black line in Fig. 4E and Fig. S10A,B). Using this threshold, more than 80% of all fate decisions could be correctly predicted based on the GATA4-mCherry classifier (Fig. S10C). This predictability of fate choice by GATA4-mCherry expression levels suggests this transcription factor is a dominant input into the decision in ESCs.

### FGF/MAPK signaling modulates the proportion of cells with PrE-like differentiation

**Fig. 5.**
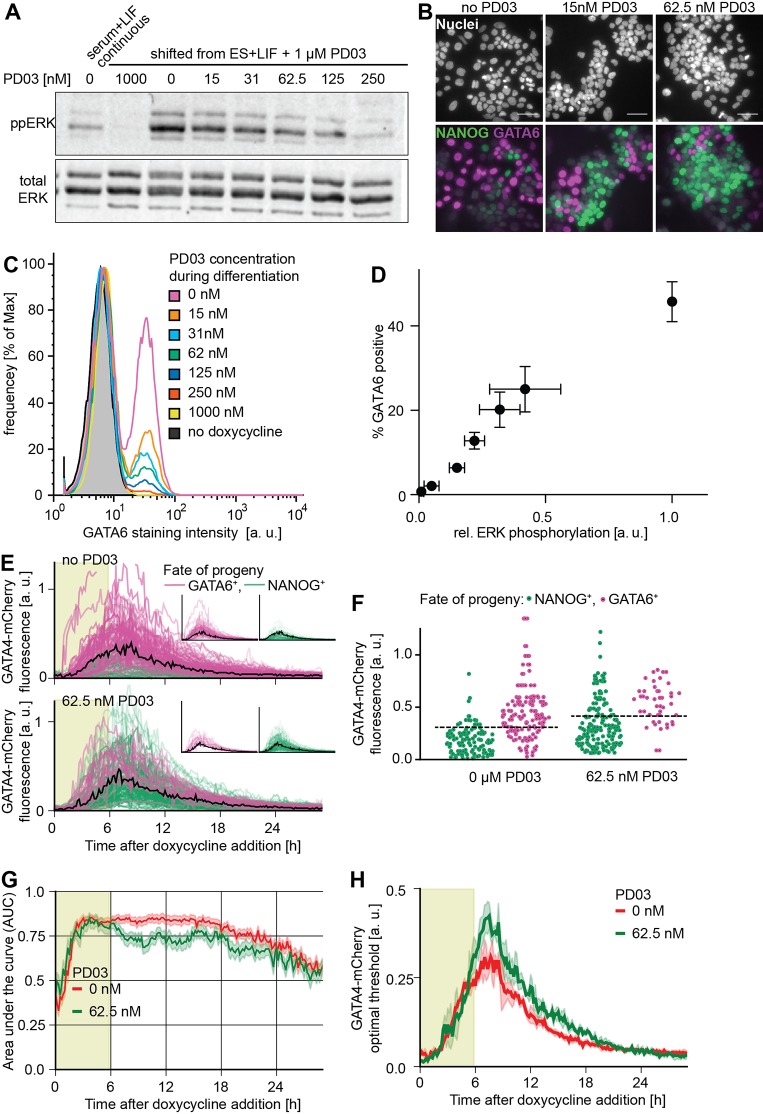
**MAPK signaling controls the proportion of cells with PrE-like differentiation.** (A) Immunoblot detecting phosphorylated ERK (top) and total ERK (bottom) in cells grown for 3 days in the presence of serum and 1 µM PD03 1 h after transfer into medium containing indicated concentrations of PD03. (B) Immunostaining of ESCs after a 6 h pulse of doxycycline-induced GATA4-mCherry expression and 24 h of differentiation in the indicated concentrations of PD03. Scale bars: 50 µm. (C) Flow cytometry of GATA6 expression in cells treated as in B. (D) Plot of relative Erk phosphorylation levels versus percentage of GATA6-positive cells for different concentrations of PD03. Data points show mean±s.d. from three independent experiments per condition. (E) GATA4-mCherry fluorescence traces of cells filmed during and after a 6 h doxycycline pulse in the absence of PD03 (top) or with 62.5 nM PD03 (bottom). Color-code of individual traces is informed by immunostaining for NANOG and GATA6 at the end of the time-lapse. Light green shaded area indicates presence of doxycycline, black trace indicates optimal threshold estimated by ROC, and insets show traces for cells with GATA6- and NANOG-positive progeny separately. (F) GATA4-mCherry fluorescence intensity of cells from the experiment shown in E at a single time-point 1 h after removal of doxycycline in the absence of PD03 (left) and in 62.5 nM PD03 (right). (G) AUC values determined by ROC analysis of the dataset shown in E for no PD03 (red) and 62.5 nM PD03 (green). AUC values decay after ∼20 h as a result of the very low levels of GATA4-mCherry expression at the end of this experiment. Error margin indicates s.d. determined by bootstrapping (*n*=1000). (H) The optimal threshold to predict differentiation from GATA4-mCherry expression in the absence of PD03 (red) and in the presence of 62.5 nM PD03 (green) increases with decreasing MAPK signaling. Error margins indicate s.d. determined by bootstrapping (*n*=1000).

In the mouse embryo, both GATA factors and FGF/MAPK signaling are required to establish PrE identity. Having shown above that inhibiting MAPK signaling prior to doxycycline-induced GATA expression increases the proportion of cells with PrE-like differentiation, we next wanted to test how MAPK signaling affected the decision to embark on PrE-like differentiation during and after the GATA pulse. MAPK activity required for PrE-like differentiation was almost completely saturated in serum-free medium, possibly through autocrine FGF signaling (Figs S11, S12), prompting us to tune the levels of Erk phosphorylation following removal of the pre-culture medium with subsaturating doses of PD03 (Fig. 5A). Partial inhibition of MAPK signaling during the 6 h doxycycline pulse and the 24 h chase period reduced the fraction of GATA6-positive cells, but not the expression levels of GATA6 in individual differentiated cells (Fig. 5B,C), with a quasi-linear relationship between the level of Erk phosphorylation and the percentage of differentiating cells (Fig. 5D). We obtained similar results using the FGF receptor inhibitor PD173074 (Fig. S12), indicating that most of the MAPK activity relevant for PrE-like differentiation of ESCs is triggered by FGF ligands, consistent with literature reports (Kunath et al., 2007). FGF/MAPK signaling levels therefore control the fraction of cells that embark on the PrE-like differentiation path.

To investigate how partial MEK inhibition affected the GATA4-mCherry threshold required for PrE-like differentiation, we performed time-lapse imaging and cell tracking for maximal and reduced MAPK signaling in parallel (Fig. 5E,F), using a PD03 concentration that led to a significant reduction of the number of differentiating cells without inducing cell death (Movie 3). ROC analysis gave similar AUC values for both conditions, indicating that differentiation can be predicted based on GATA4-mCherry expression levels with similar confidence at different signaling levels (Fig. 5G). However, the optimal prediction threshold was consistently increased upon partial MEK inhibition (Fig. 5F,H). We conclude that MAPK signaling levels set the GATA4-mCherry threshold dose required to trigger differentiation.

We noticed that the distribution of GATA4-mCherry expression levels in differentiating and non-differentiating cells changed upon partial inhibition of signaling (Fig. 5E,F). In addition to setting the transcription factor threshold dose, partial MEK inhibition therefore appears to modulate heterogeneities in the population that affect PrE-like differentiation.

### A simple mutual repression circuit recapitulates the experimentally observed gene expression dynamics

**Fig. 6.**
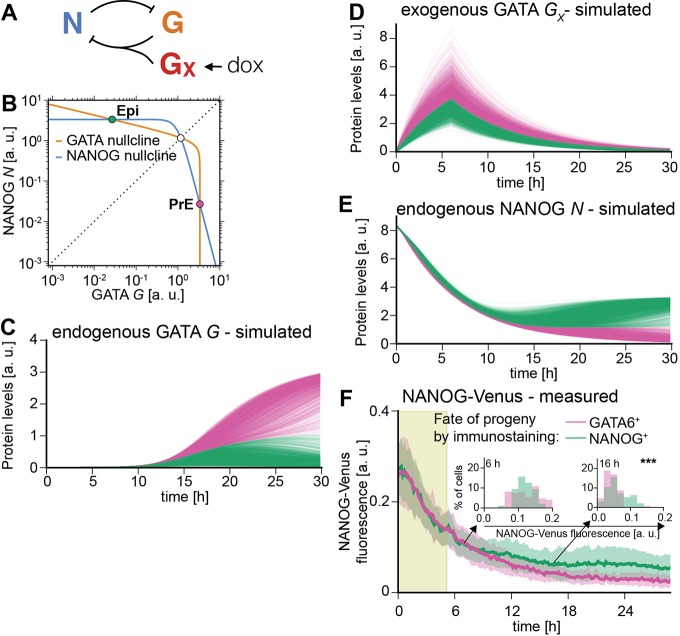
**A simple mutual repression circuit recapitulates experimentally observed expression dynamics.** (A) Connectivity of the mutual repression circuit used in dynamic simulations. (B) Phase portrait depicting the autonomous dynamics of the system in A. Intersections of the NANOG nullcline (blue) and the GATA nullcline (orange) define three steady states, two of which are stable and correspond to the NANOG^high^, GATA^low^ Epi state (green dot) or the NANOG^low^, GATA^high^ PrE state (purple). (C-E) Simulated time traces of endogenous GATA *G* (C), exogenous GATA4-mCherry *G_X_* (D), and NANOG *N* expression (E). Color code is informed by final GATA expression levels in C (GATA^high^, purple; GATA^low^, green), and corresponding traces have same color in C, D and E. (F) Average NANOG-Venus expression levels in cells carrying a NANOG-Venus translational reporter during and after 6 h of doxycycline-induced GATA4-mCherry expression. Cells were classified as GATA6-positive and NANOG-positive by immunostaining after the time-lapse. Presence of doxycycline is indicated by light green shading. Purple and green lines and shaded areas indicate mean NANOG-Venus fluorescence levels±s.d. Insets show histograms for distributions of NANOG-Venus fluorescence in the two classes of cells at 6 h and 16 h after the start of recording; ***P≤0.0001 (Mann–Whitney U-test). See also Movie 4.

To gain insights into the formal nature of the interactions between signaling and transcriptional regulators, we then sought to identify the minimal circuit model of the components of the decision machinery that would recapitulate our data. The irreversible, switch-like behavior of our system indicates the presence of positive feedback in the underlying genetic network. Because NANOG directly represses *Gata6* (Singh et al., 2007), and GATA expression led to rapid repression of NANOG expression in our system, we chose a network of two mutually repressive nodes, GATA and NANOG, as a minimal system with net positive feedback to formalize a bistable genetic switch (Cherry and Adler, 2000; Plahte et al., 1995; Snoussi, 1998; Thomas, 1981) (Fig. 6A; see supplementary materials and methods for a detailed description of the model). This system is described by two coupled ordinary differential equations that account for the dynamics of NANOG (*N*) and endogenous GATA (*G*) as markers for the Epi and PrE programs in individual cells, respectively:
(1)
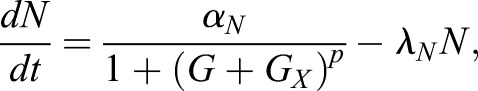

(2)
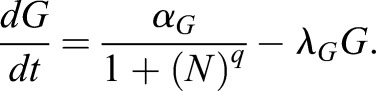

A third equation models the externally supplied pulse of GATA (*G_X_*) that drives the endogenous circuit:
(3)
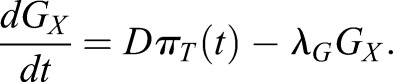
To reflect the experimentally observed heterogeneous expression of exogenous GATA factors, we varied the maximum transcription rate *D* of exogenous GATA between cells (for details see the supplementary materials and methods)*_._* This was the only source of cell-to-cell variability in our model. As initial conditions, we endowed cells with high levels of NANOG and no GATA to reflect pre-culturing in the presence of PD03.

To assess the dynamics of the endogenous circuit described by this model we plotted the nullclines *dG/dt=0* and *dN/dt=0* for the specific set of parameters used*.* Two of the three equilibrium states defined by the intersections of the nullclines are stable and correspond to the fully differentiated GATA-positive and the undifferentiated NANOG-positive state, respectively (Fig. 6B). A boundary in the phase space (dashed line in Fig. 6B) separates the combinations of GATA and NANOG levels which will evolve into the fully differentiated GATA-positive state from those that lead to the undifferentiated NANOG-positive state. To induce PrE-like differentiation, the exogenous GATA input has to exceed the threshold required to move cells across this boundary by sufficiently repressing NANOG and allowing for endogenous GATA expression. For the chosen parameter set, simulated time traces of individual cells closely resembled the experimentally observed expression dynamics of the endogenous *Gata6* gene (Fig. 6C, compare with Fig. 3F), and exogenous GATA4-mCherry (Fig. 6D, compare with Fig. 4E), suggesting this simple mutual repression circuit is sufficient to capture essential dynamics of the experimental system.

To further test the model, we compared the dynamics of NANOG expression *in silico* and *in vivo*. In model simulations, NANOG expression levels first decreased rapidly in all cells from the initial conditions chosen to represent the effects of the pre-culture regime towards lower steady state levels, before differences in NANOG expression levels in differentiating and non-differentiating cells became apparent (Fig. 6E). To monitor NANOG expression dynamics experimentally, we integrated a previously described NANOG-Venus translational fusion reporter (Filipczyk et al., 2013) into the inducible cell line. Following transient GATA4-mCherry expression, this reporter showed expression dynamics that were in close agreement with the model simulations, further supporting the idea that a simple mutual repression circuit is sufficient to capture the dynamics of the system.

### Inhibition of the Epi program by MAPK signaling controls the proportion of cells with PrE-like differentiation

**Fig. 7.**
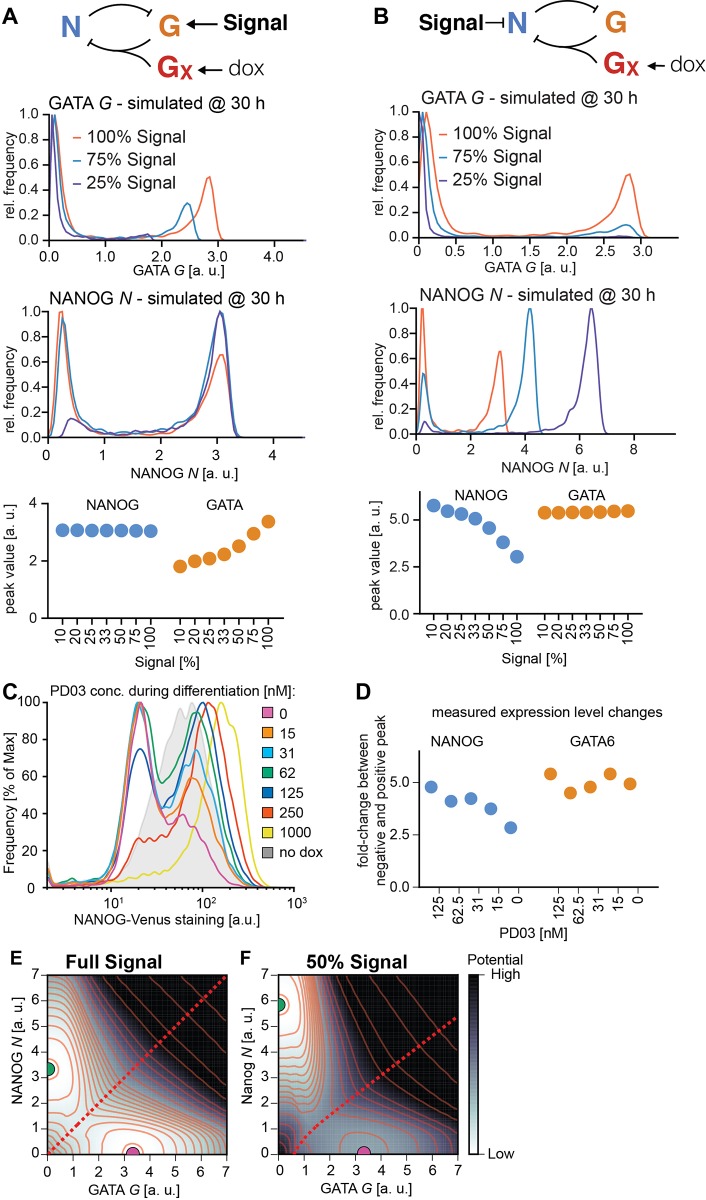
**MAPK signaling controls the proportion of PrE-like cells through inhibition of the Epi program.** (A,B) Top panels: modes of signaling interactions tested in the model. (A) Signaling promotes GATA production; (B) signaling inhibits NANOG production. Middle panels: simulated histograms for GATA and NANOG expression at varying signaling levels (*t*=30 h; *n*=2000). Bottom panels: location of high peaks in the above histograms. (C) Flow cytometry of NANOG-Venus reporter cells stained with antibodies directed against the Venus tag 30 h after a 6 h doxycycline pulse. This method detected a bimodal distribution of NANOG-Venus-positive and -negative cells with a higher signal-to-noise ratio than direct staining for NANOG. Traces are smoothened to show positions of peaks more clearly. (D) Fold-change of the NANOG-Venus-positive (blue) and GATA6-positive (orange) peaks relative to the respective negative peaks estimated from the flow cytometry experiments in C (NANOG-Venus) and Fig. 5C (GATA6) for different concentrations of PD03. See Fig. S13 for details on estimation of peak positions. (E,F) Quasi-potentials derived from the autonomous dynamics of the system in B for two signaling levels. Darker regions have higher potential, i.e. faster dynamic changes. The dashed red line indicates the separatrix (ridge) separating the basins of attraction of the NANOG^high^, GATA^low^ Epi fate (green dot) and the NANOG^low^, GATA^high^ PrE fate (purple dot). Lowered signaling (F) shifts and bends the separatrix, thereby changing the relative sizes of the two basins of attraction.

To pinpoint the main mechanism by which FGF/MAPK signaling controls the fraction of cells with PrE-like differentiation, we considered two simple extensions of the model, one in which FGF/MAPK signaling promotes expression of the PrE program (Fig. 7A), and an alternative model in which signaling inhibits the Epi program (Fig. 7B). In both cases a reduction of signaling led to a simulated increase in the number of cells in the NANOG-positive peak and a decrease of cells in the GATA-positive peak (Fig. 7A,B, middle). However, expression levels in the respective positive peaks changed in distinct ways depending on the type of signaling input (Fig. 7A,B, middle and bottom). Estimating changes in GATA6 and NANOG expression levels upon partial MEK inhibition from flow cytometry data showed that GATA6 expression levels in individual cells remained approximately constant in the presence of different doses of PD03, whereas the NANOG-Venus positive peak consistently shifted to higher expression levels with lowered signaling (Fig. 7C,D; Fig. S13). While not ruling out a more complex integration of FGF/MAPK signaling into the regulatory circuit underlying PrE-like differentiation, these results suggest that a major route by which FGF/MAPK signaling controls the fraction of cells with PrE-like differentiation is through inhibition of the Epi-specific gene expression program. This conclusion is further supported by a recent report showing direct inhibition of NANOG expression by FGF/MAPK signaling mediated by chromatin modifications (Hamilton and Brickman, 2014).

Finally, we sought to develop a visual representation of the system's dynamics in different signaling regimes by estimating the path-integral quasi-potential surfaces (Bhattacharya et al., 2011) of the system for two different signaling levels (Fig. 7E,F). This representation highlights two basins of attraction corresponding to the NANOG-positive state and the GATA-positive state, respectively. A reduction in signaling bends the ridge that separates the basins towards the GATA-positive state, making its basin of attraction narrower and shallower relative to that of the NANOG-positive state (Fig. 7E,F).

We conclude that a simple mutual repression circuit is sufficient to capture the dynamic hallmarks of the Epi-versus-PrE fate decision, and that through repression of the Epi program, FGF/MAPK signaling sets the relative sizes of the basins of attraction corresponding to the two fates defined by this circuit, allowing signaling to regulate the proportions of cells adopting either fate.

## DISCUSSION

Here we have used engineered mouse ESC lines to study the mechanism underlying the decision between the Epi and the PrE fate. Our experimental system allowed us to modulate and measure quantitatively the transcription factor and signaling inputs into the decision, and to follow the dynamics of the decision at the level of single cells. We have uncovered two successive functions of MAPK signaling in the ESC system. Before the induced expression of GATA factors, inhibition of MAPK signaling is required to make the PrE-like differentiation program accessible in ESCs. Once exogenous GATA factors are expressed, MAPK signaling is required to execute the decision of PrE-like differentiation. The Epi-versus-PrE differentiation event displays hallmarks of an irreversible bistable switch, as co-expression of determinants of the Epi and the PrE fate resolves into one of two mutually exclusive stable states characterized by NANOG and GATA expression, respectively. We detect a well-defined threshold level of exogenous GATA factor expression required to flip this switch and induce differentiation, and find that MAPK signaling sets this threshold dose. This decision is therefore a strongly regulated process that is largely determined by few well-defined transcriptional and signaling inputs.

### The accessibility of the PrE program depends on ESC culture conditions

We find that cells cultured in the presence of serum are refractory to PrE-like differentiation upon induced GATA expression, but responsiveness to doxycycline-induced GATA factors can be restored by extended exposure to GSK3 or MEK inhibitors, e.g. in 2i medium. One interpretation of this finding is that ESCs cultured in serum are strongly biased towards embryonic fates, and as a consequence have blocked the PrE-like differentiation program. In line with this idea, ESCs grown in the presence of serum display higher levels of repressive chromatin marks on a subset of promoters, including the *Gata6* promoter, than cells grown in 2i+LIF (Marks et al., 2012). Furthermore, the transcriptome of ICM cells resembles more closely that of ESCs cultured in 2i medium than that of ESCs cultured in serum (Boroviak et al., 2014). This suggests that pre-culture in 2i brings ESCs to a molecular state mirroring that of early ICM cells, from which, upon induced GATA expression, the decision between the Epi and the PrE fate can be taken similarly to the situation in the embryo.

### Extraembryonic fate choice is determined by the output of a simple mutual repression circuit

Our finding that precise measurements of GATA4-mCherry expression levels allow prediction of fate decisions in individual ESCs with high confidence before endogenous fate markers appear led us to formulate a minimal genetic circuit model with deterministic regulation to formalize the mechanism of the decision process. Our model solely consists of mutually repressive interactions between the Epi- and the PrE-like program, modulated by a repressive input of FGF/MAPK on the Epi program (Fig. 7B). This is sufficient to recapitulate the experimentally observed dynamics of lineage marker expression, to model bistable behavior, and to formalize our finding that the role of MAPK signaling is to set a GATA threshold required for PrE-like differentiation. Our minimal model is a subnetwork of a more complex model for the Epi-versus-PrE fate decision recently developed by Bessonnard et al. (2014). Bessonnard's model posits an additional positive input of FGF/MAPK signaling onto GATA expression, and contains positive autoregulatory feedback loops centered on both NANOG and GATA, which endow the dynamic system with a third stable state of NANOG and GATA co-expression. This allowed Bessonnard et al. to simulate both the establishment and the resolution of the co-expression state in a single model. Focusing on the resolution of the co-expression state, our data suggest that the additional links of Bessonnard's model are not required to explain the dynamics of this phase of the decision. It remains, however, possible that positive autoregulation of the Epi and PrE programs and a positive input of FGF/MAPK signaling on GATA expression fine-tune the response of cells during this stage of the decision process.

We note that not all cells abide by the GATA4-mCherry threshold that best predicts PrE-like differentiation. This might reflect persistent heterogeneous chromatin configurations that block PrE-like differentiation in individual cells, or be a consequence of heterogeneous MAPK signaling among ESCs. Signaling heterogeneities have been detected in other cell lines (Albeck et al., 2013; Aoki et al., 2013), and we expect they will be functionally relevant for PrE-like differentiation of ESCs.

### Integration of signaling into the mutual repression circuit serves to balance proportions of cell fates in developing tissues

The mathematical model of a mutual repression circuit has previously been applied to describe the dynamics of the switch between the lysogenic and lytic phases of the lifecycle of bacteriophage lambda (Ptashne, 2004), and a genetically engineered toggle switch circuit in *Escherichia* *coli* (Gardner et al., 2000). Our work is one of the first experimentally supported examples demonstrating that this network can be used to formalize the decision between two fates during mammalian development. Extending the model with a signaling input allows for dynamic control of the sizes of the basins of attraction corresponding to the different states of the bistable system. The mammalian preimplantation embryo might harness this property to balance the proportion of Epi and PrE cells. The initial expression of transcriptional regulators driving lineage choice is stochastic, possibly as a consequence of the mechanisms that control gene expression in the early embryo (Dietrich and Hiiragi, 2007; Ohnishi et al., 2014). The resulting heterogeneous distributions of transcription factor concentrations will bias ICM cells towards specific fates (Xenopoulos et al., 2015). It has been shown that lineage commitment occurs non-synchronously in the cells of the ICM, and that the first cells to commit are fated towards the epiblast (Grabarek et al., 2012). Because Epi cells produce FGF4 (Frankenberg et al., 2011; Nichols et al., 1998), FGF4 levels will reflect the number of Epi-committed cells and act on the as yet uncommitted cells. By modulating the bistable switch operating in these cells, this process might ultimately place the appropriate number of uncommitted cells in the basin of attraction corresponding to the PrE fate. FGF/MAPK signaling might thus acts as a feedback mechanism to balance proportions of two distinct cell fates in populations (Lander et al., 2009). It will be interesting to see whether this new principle applies to differentiation decisions beyond those in the preimplantation embryo.

## MATERIALS AND METHODS

### ESC culture and genetic manipulation

For genetic engineering, ESCs were grown on mitotically inactivated mouse embryonic fibroblasts in Knockout DMEM (Gibco) supplemented with 15% fetal bovine serum (FBS), 50 µM β-mercaptoethanol, glutamax, non-essential amino acids and 1 µg/ml LIF. After line derivation, feeders were removed by serial passaging, and cells were maintained on gelatin-coated dishes in GMEM-based medium supplemented with 10% FBS, sodium pyruvate, 50 µM β-mercaptoethanol, glutamax, non-essential amino acids and LIF.

Serum-free media were based on N2B27 (NDiff 227, Stem Cells) and supplemented with 3 µM CHIR99021, 1 µM PD0325901 and 1 µg/ml LIF to give 2i+LIF. For the experiments described in Fig. S11, N2B27 was supplemented with 10 ng/ml BMP4 (R&D), 1 µg/ml LIF and 1 µg/ml heparin.

All cell lines used in this study were based on the KH2 ESC line (Beard et al., 2006). Engineering of ESCs is described in more detail in the supplementary materials and methods. Transgene expression was induced by adding 500 ng/ml doxycycline to the culture medium.

### Immunocytochemistry

Cells for immunocytochemistry were grown on ibidi µ-slides and stained as described in Kalmar et al. (2009). Primary antibodies were anti-NANOG (1:200; eBiosciences, 14-5761), anti-FLAG (1:1000; Sigma M2, F3165), anti-GATA6 (1:200; R&D, AF1700) and anti-GATA4 (1:200; Santa Cruz, sc-9053). Detection was performed using Alexa Fluor-conjugated secondary antibodies at 4 μg/ml (Molecular Probes). Nuclei were visualized using Hoechst 33342 dye at 100 μg/ml (Molecular Probes, H1399). Cells were imaged on a Zeiss LSM700 confocal microscope with a 40× oil immersion lens (NA 1.3).

### Flow cytometry

Cells for flow cytometry were trypsinized and either analyzed immediately or fixed for 15 min in 3% PFA/PBS. Intracellular antigens were stained in suspension using the same primary and secondary antibodies as used for immunostaining. mCherry fluorescence was measured on a BD Fortessa Flow cytometer, all other flow cytometric analysis was performed using a Beckman Coulter CyAn ADP analyzer. Cell sorting was done on a Beckman Coulter MoFlo. To estimate peak positions, histograms were smoothened, followed by detection of local maxima with custom-written Python scripts.

### Immunoblotting

For immunoblotting, cells were lysed in RIPA buffer and lysates were separated on polyacrylamide gels before transfer to nitrocellulose membranes. Antibodies used were anti-ppErk (Sigma, M9692) and anti-Erk1/2 (Millipore, 06-182) at 1:500 dilution. Detection was performed using fluorescently labeled secondary antibodies at 0.1 μg/ml (LI-COR) and scanning in a LI-COR Odyssey system. Intensities of bands were quantified in ImageStudio (LI-COR).

### Time-lapse imaging and cell tracking

Time-lapse imaging was performed in DMEM-based medium without Phenol Red, supplemented as detailed above. We used a Zeiss Axiovert M200 microscope equipped with a SOLA LED light source, an Andor iXON Ultra 888 EMCCD camera and a heated stage with CO_2_ supply. Hardware was controlled by MicroManager software (Edelstein et al., 2001). Time-lapse movies were acquired using a 40× long-working distance lens. See the supplementary materials and methods for details on image analysis.

### Mathematical modeling

Numerical simulations of the model were implemented in Python language. Parameter values used in the simulations are given in Table S1. For details on the model see the supplementary materials and methods.

## References

[DEV127530C1] Albeck J. G., Mills G. B. and Brugge J. S. (2013). Frequency-modulated pulses of ERK activity transmit quantitative proliferation signals. *Mol. Cell* 49, 249-261. 10.1016/j.molcel.2012.11.002 23219535 PMC4151532

[DEV127530C2] Aoki K., Kumagai Y., Sakurai A., Komatsu N., Fujita Y., Shionyu C. and Matsuda M. (2013). Stochastic ERK activation induced by noise and cell-to-cell propagation regulates cell density-dependent proliferation. *Mol. Cell* 52, 529-540. 10.1016/j.molcel.2013.09.015 24140422

[DEV127530C3] Beard C., Hochedlinger K., Plath K., Wutz A. and Jaenisch R. (2006). Efficient method to generate single-copy transgenic mice by site-specific integration in embryonic stem cells. *Genesis* 44, 23-28. 10.1002/gene.20180 16400644

[DEV127530C4] Beddington R. S. and Robertson E. J. (1989). An assessment of the developmental potential of embryonic stem cells in the midgestation mouse embryo. *Development* 105, 733-737. 2598811 10.1242/dev.105.4.733

[DEV127530C5] Bessonnard S., De Mot L., Gonze D., Barriol M., Dennis C., Goldbeter A., Dupont G. and Chazaud C. (2014). Gata6, NANOG and Erk signaling control cell fate in the inner cell mass through a tristable regulatory network. *Development* 141, 3637-3648. 10.1242/dev.109678 25209243

[DEV127530C6] Bhattacharya S., Zhang Q. and Andersen M. E. (2011). A deterministic map of Waddington's epigenetic landscape for cell fate specification. *BMC Syst. Biol.* 5, 85 10.1186/1752-0509-5-85 21619617 PMC3213676

[DEV127530C7] Boroviak T., Loos R., Bertone P., Smith A. and Nichols J. (2014). The ability of inner-cell-mass cells to self-renew as embryonic stem cells is acquired following epiblast specification. *Nat. Cell Biol.* 16, 516-528. 10.1038/ncb2965 24859004 PMC4878656

[DEV127530C8] Chambers I., Silva J., Colby D., Nichols J., Nijmeijer B., Robertson M., Vrana J., Jones K., Grotewold L. and Smith A. (2007). NANOG safeguards pluripotency and mediates germline development. *Nature* 450, 1230-1234. 10.1038/nature06403 18097409

[DEV127530C9] Cherry J. L. and Adler F. R. (2000). How to make a biological switch. *J. Theor. Biol.* 203, 117-133. 10.1006/jtbi.2000.1068 10704297

[DEV127530C10] Cho L. T. Y., Wamaitha S. E., Tsai I. J., Artus J., Sherwood R. I., Pedersen R. A., Hadjantonakis A.-K. and Niakan K. K. (2012). Conversion from mouse embryonic to extra-embryonic endoderm stem cells reveals distinct differentiation capacities of pluripotent stem cell states. *Development* 139, 2866-2877. 10.1242/dev.078519 22791892 PMC3403099

[DEV127530C11] Dietrich J.-E. and Hiiragi T. (2007). Stochastic patterning in the mouse pre-implantation embryo. *Development* 134, 4219-4231. 10.1242/dev.003798 17978007

[DEV127530C12] Edelstein A., Amodaj N., Hoover K., Vale R. and Stuurman N. (2001). *Computer Control of Microscopes Using µManager*. Hoboken, NJ, USA: John Wiley & Sons. 10.1002/0471142727.mb1420s92 PMC3065365 20890901

[DEV127530C13] Fawcett T. (2006). An introduction to ROC analysis. *Pattern Recognit. Lett.* 27, 861-874. 10.1016/j.patrec.2005.10.010

[DEV127530C14] Filipczyk A., Gkatzis K., Fu J., Hoppe P. S., Lickert H., Anastassiadis K. and Schroeder T. (2013). Biallelic expression of NANOG protein in mouse embryonic stem cells. *Cell Stem Cell* 13, 12-13. 10.1016/j.stem.2013.04.025 23827706

[DEV127530C15] Frankenberg S., Gerbe F., Bessonnard S., Belville C., Pouchin P., Bardot O. and Chazaud C. (2011). Primitive endoderm differentiates via a three-step mechanism involving NANOG and RTK signaling. *Dev. Cell* 21, 1005-1013. 10.1016/j.devcel.2011.10.019 22172669

[DEV127530C42] Freyer L., Schröter C., Saiz N., Schrode N., Nowotschin S., Martinez Arias A. and Hadjantonakis A.-K. (2015). A loss-of-function and H2B-Venus transcriptional reporter allele for Gata6 in mice. *BMC Dev. Biol.* 15, 38 10.1186/s12861-015-0086-5 26498761 PMC4619391

[DEV127530C16] Fujikura J., Yamato E., Yonemura S., Hosoda K., Masui S., Nakao K., Miyazaki J.-I. and Niwa H. (2002). Differentiation of embryonic stem cells is induced by GATA factors. *Genes Dev.* 16, 784-789. 10.1101/gad.968802 11937486 PMC186328

[DEV127530C17] Gardner T. S., Cantor C. R. and Collins J. J. (2000). Construction of a genetic toggle switch in *Escherichia coli*. *Nature* 403, 339-342. 10.1038/35002131 10659857

[DEV127530C18] Grabarek J. B., Zyzynska K., Saiz N., Piliszek A., Frankenberg S., Nichols J., Hadjantonakis A.-K. and Plusa B. (2012). Differential plasticity of epiblast and primitive endoderm precursors within the ICM of the early mouse embryo. *Development* 139, 129-139. 10.1242/dev.067702 22096072 PMC3231774

[DEV127530C19] Hamilton W. B. and Brickman J. M. (2014). Erk signaling suppresses embryonic stem cell self-renewal to specify endoderm. *Cell Rep.* 9, 2056-2070. 10.1016/j.celrep.2014.11.032 25533345

[DEV127530C20] Kalmar T., Lim C., Hayward P., Muñoz-Descalzo S., Nichols J., Garcia-Ojalvo J. and Martinez Arias A. (2009). Regulated fluctuations in NANOG expression mediate cell fate decisions in embryonic stem cells. *PLoS Biol.* 7, e1000149 10.1371/journal.pbio.1000149 19582141 PMC2700273

[DEV127530C21] Kang M., Piliszek A., Artus J. and Hadjantonakis A.-K. (2012). FGF4 is required for lineage restriction and salt-and-pepper distribution of primitive endoderm factors but not their initial expression in the mouse. *Development* 140, 267-279. 10.1242/dev.084996 23193166 PMC3597205

[DEV127530C22] Kunath T., Saba-El-Leil M. K., Almousailleakh M., Wray J., Meloche S. and Smith A. (2007). FGF stimulation of the Erk1/2 signalling cascade triggers transition of pluripotent embryonic stem cells from self-renewal to lineage commitment. *Development* 134, 2895-2902. 10.1242/dev.02880 17660198

[DEV127530C23] Lander A. D., Gokoffski K. K., Wan F. Y. M., Nie Q. and Calof A. L. (2009). Cell lineages and the logic of proliferative control. *PLoS Biol.* 7, e1000015 10.1371/journal.pbio.1000015 PMC2628408 19166268

[DEV127530C24] Marks H., Kalkan T., Menafra R., Denissov S., Jones K., Hofemeister H., Nichols J., Kranz A., Francis Stewart A., Smith A. et al. (2012). The transcriptional and epigenomic foundations of ground state pluripotency. *Cell* 149, 590-604. 10.1016/j.cell.2012.03.026 22541430 PMC3398752

[DEV127530C25] Morgani S. M., Canham M. A., Nichols J., Sharov A. A., Migueles R. P., Ko M. S. H. and Brickman J. M. (2013). Totipotent embryonic stem cells arise in ground-state culture conditions. *Cell Rep.* 3, 1945-1957. 10.1016/j.celrep.2013.04.034 23746443 PMC3701323

[DEV127530C26] Mulvey C. M., Schröter C., Gatto L., Dikicioglu D., Fidaner I. B., Christoforou A., Deery M. J., Cho L. T. Y., Niakan K. K., Martinez Arias A. et al. (2015). Dynamic proteomic profiling of extra-embryonic endoderm differentiation in mouse embryonic stem cells. *Stem Cells* 33, 2712-2725. 10.1002/stem.2067 26059426

[DEV127530C27] Nichols J., Zevnik B., Anastassiadis K., Niwa H., Klewe-Nebenius D., Chambers I., Schöler H. and Smith A. (1998). Formation of pluripotent stem cells in the mammalian embryo depends on the POU transcription factor Oct4. *Cell* 95, 379-391. 10.1016/S0092-8674(00)81769-9 9814708

[DEV127530C28] Nichols J., Silva J., Roode M. and Smith A. (2009). Suppression of Erk signalling promotes ground state pluripotency in the mouse embryo. *Development* 136, 3215-3222. 10.1242/dev.038893 19710168 PMC2739140

[DEV127530C29] Ohnishi Y., Huber W., Tsumura A., Kang M., Xenopoulos P., Kurimoto K., Oleś A. K., Araúzo-Bravo M. J., Saitou M., Hadjantonakis A.-K., et al. (2014). Cell-to-cell expression variability followed by signal reinforcement progressively segregates early mouse lineages. *Nat. Cell Biol.* 16, 27-37. 10.1038/ncb2881 24292013 PMC4062977

[DEV127530C30] Plahte E., Mestl T. and Omholt S. W. (1995). Feedback loops, stability and multistationarity in dynamical systems. *J. Biol. Syst.* 3, 409-413. 10.1142/S0218339095000381

[DEV127530C31] Plusa B., Piliszek A., Frankenberg S., Artus J. and Hadjantonakis A.-K. (2008). Distinct sequential cell behaviours direct primitive endoderm formation in the mouse blastocyst. *Development* 135, 3081-3091. 10.1242/dev.021519 18725515 PMC2768606

[DEV127530C32] Ptashne M. (2004). *A Genetic Switch*. Cold Spring Harbor: CSHL Press.

[DEV127530C33] Rossant J. and Tam P. P. L. (2009). Blastocyst lineage formation, early embryonic asymmetries and axis patterning in the mouse. *Development* 136, 701-713. 10.1242/dev.017178 19201946

[DEV127530C34] Schrode N., Saiz N., Di Talia S. and Hadjantonakis A.-K. (2014). GATA6 Levels modulate primitive endoderm cell fate choice and timing in the mouse blastocyst. *Dev. Cell* 29, 454-467. 10.1016/j.devcel.2014.04.011 24835466 PMC4103658

[DEV127530C35] Shimosato D., Shiki M. and Niwa H. (2007). Extra-embryonic endoderm cells derived from ES cells induced by GATA factors acquire the character of XEN cells. *BMC Dev. Biol.* 7, 80 10.1186/1471-213X-7-80 PMC1933422 17605826

[DEV127530C36] Singh A. M., Hamazaki T., Hankowski K. E. and Terada N. (2007). A heterogeneous expression pattern for NANOG in embryonic stem cells. *Stem Cells* 25, 2534-2542. 10.1634/stemcells.2007-0126 17615266

[DEV127530C37] Snoussi E. H. (1998). Necessary conditions for multistationarity and stable periodicity. *J. Biol. Syst.* 6, 3-9. 10.1142/S0218339098000042

[DEV127530C38] Thomas R. (1981). On the relation between the logical structure of systems and their ability to generate multiple steady states or sustained oscillations. In *Springer Series in Synergetics* (ed. Dora D. J., Demongeot J. and Lacolle B.), pp. 180-193. Berlin: Springer.

[DEV127530C39] Wamaitha S. E., del Valle I., Cho L. T. Y., Wei Y., Fogarty N. M. E., Blakeley P., Sherwood R. I., Ji H. and Niakan K. K. (2015). Gata6 potently initiates reprograming of pluripotent and differentiated cells to extraembryonic endoderm stem cells. *Genes Dev.* 29, 1239-1255. 10.1101/gad.257071.114 26109048 PMC4495396

[DEV127530C40] Xenopoulos P., Kang M., Puliafito A., Di Talia S. and Hadjantonakis A.-K. (2015). Heterogeneities in NANOG expression drive stable commitment to pluripotency in the mouse blastocyst. *Cell Rep.* 10, 1508-1520. 10.1016/j.celrep.2015.02.010 PMC4560681 25753417

[DEV127530C41] Yamanaka Y., Lanner F. and Rossant J. (2010). FGF signal-dependent segregation of primitive endoderm and epiblast in the mouse blastocyst. *Development* 137, 715-724. 10.1242/dev.043471 20147376

